# The role of patient and public involvement in rapid qualitative studies: Can we carry out meaningful PPIE with time pressures?

**DOI:** 10.1186/s40900-022-00402-5

**Published:** 2022-11-30

**Authors:** Katie Gilchrist, Syka Iqbal, Cecilia Vindrola-Padros

**Affiliations:** 1 Department of Targeted Intervention, University College London, London, UK; 2 Department of Psychology, University of Bradford, Bradford, UK

**Keywords:** PPIE, Patient and public involvement, Patient and public engagement, Rapid research, Qualitative research

## Abstract

**Introduction:**

Rapid qualitative studies conducted with patient and public involvement can help promote policy-relevant and efficient research. There is a need to understand the experiences of researchers, patients, and members of the public to guide the development of good practice and to determine the extent to which rapid qualitative research can be implemented in PPIE projects.

**Methods:**

We conducted a qualitative study to explore the experiences of research teams that carried out studies using rapid techniques with patient and public involvement. We carried out 26 interviews with researchers, coordinators, patients, carers, service users and members of the public.

**Results:**

This study identified needs which related to practical and time constraints. Rapid qualitative research tends to be limited to certain PPIE groups, and particular phases of the research process. Study findings are rarely discussed with PPIE members. The educational needs of rapid qualitative research were also identified. Researchers and PPIE members considered three main issues: a lack of training on patient involvement for researchers, rapid qualitative research training for PPIE members, and the diversity of PPIE members.

**Conclusion:**

We found that rapid researchers were able to involve patients and members of the public in research despite time pressures. The challenges identified in this study can be used to plan future training programmes for researchers and PPIE panel members and develop strategies to recruit PPIE panel members from a wide range of backgrounds.

**Public contribution:**

The research aim was to explore the experiences of those carrying out rapid qualitative studies with PPIE. As such, the participants of this study included patients, carers, service users and members of the public, who were interviewed individually. A lived experienced researcher and PPIE member for a hospital conducted the design, data collection and analysis of the study. The study brief was to interview researchers only. The lived-experience researcher initiated the inclusion of PPIE members as participants, therefore strengthening the study design. We shared the draft report with the PPIE participants for participant validation and to maintain a continuous feedback relationship. This led to addressing key issues in designing and involving PPIE members in more meaningful and equal ways. Whilst there is agreement on activities which centre on PPIE, there is no consensus on how to achieve these in high quality rapid qualitative studies.

**Supplementary Information:**

The online version contains supplementary material available at 10.1186/s40900-022-00402-5.

## Background

Patient and public involvement in research is increasingly expected in the design, production, and dissemination of research [1–3]. Successful patient and public involvement and engagement (PPIE) approaches include those where there is equal partnership between researchers and PPIE members [4] and where patients and members of the public from a wide range of backgrounds are actively engaged in research (including those from hard-to-reach, seldom heard populations) [5–7]. Involvement differs from participation in that the patients and members of the public being involved, contribute to the research process as advisors [3]. In this paper, we argue PPIE should be ‘meaningful’ which is based on principles of valuing partnerships, cultivating learning, identifying training needs and resources of researchers and PPIE members, and being responsive to patients and public members [8]. Researchers have argued that meaningful engagement is a complex process that often takes time and is based on building trust and rapport between researchers, patients, and members of the public [9, 10].

Barriers to carrying out PPIE in research, have been widely documented [10–12]. PPIE members often report they are not compensated, and the outcomes of research are rarely communicated which has led to an erosion of trust [6]. Furthermore, in cases when PPIE is attempted in time-sensitive contexts but without a pre-established model, it is improvised and becomes a tokenistic exercise and is at risk of being disingenuous [6, 9]. This leaves PPIE members feeling unsatisfied with their experience and undervalued, especially where the relationships between researchers and PPIE is fragile. For meaningful and positive partnerships for both PPIE members and researchers it is crucial for research and PPIE teams to work together where PPIE members are respected as equals, and trust is developed through an ongoing process [9]. It must be noted the requirement for long-term engagement poses difficulties for research teams using rapid approaches for research and evaluation to deliver findings at a time when these can be used to inform decision making processes and changes in healthcare practice and policy [13]. Rapid qualitative research is challenging as it involves directing research capacity, time, and funds, and is often driven by topical and pressing issues, whilst remaining iterative and inclusive. A question that often emerges is how to effectively undertake meaningful patient and public engagement in our studies, which will allow patients and members of the public to explore their expectations of their role, advise on projects, and make choices, but still implement dynamic study designs to deliver findings when these are needed.

The field of rapid research and evaluation is not new, but it has expanded considerably over the past decades to include a wide range of approaches, including rapid appraisals, rapid assessments, rapid ethnographies and rapid evaluations [13–15]. These approaches are now commonly used in healthcare contexts to generate findings within short-timeframes [14] and have been defined as: (1) studies carried out over a few weeks or a few months, (2) studies with focused or targeted research questions, (3) studies with some degree of participation from community members or other stakeholders in processes of design and/or implementation, (4) the use of iterative cycles, (5) the use of teams of researchers, and (6) the integration of feedback loops to share emerging findings with stakeholders [13, 16, 17]. Involving members of the public and patients throughout rapid qualitative research as co-producers of knowledge is fundamental and congruent with the main principles of rapid qualitative research.

Challenges in the implementation of rapid approaches in the healthcare context have been widely documented and these include the inherent tension between the use of the term ‘rapid’ and dominant assumptions that this will mean the study is of lower quality or a ‘quick and dirty exercise’, trade-offs between the breadth and depth of data, issues in the sampling of study participants and the potential risk that harder to reach, seldom heard groups will be neglected, maintaining consistency in data collection and analysis when carrying out research as a team, and the integration of critical and reflexive processes in the research process in the context of time pressures [18]. A challenge that remains unexplored is the meaningful integration of patient and public engagement in the design and implementation of rapid research and evaluation. To achieve a meaningful approach for rapid researchers, they must acquire the skills and expertise to know when to involve PPIE members, how to access and keep PPIE members involved, and acknowledge their research activities require specific training and strategies [9]. Although guidance is available [1] adapting these to specific contexts such as rapid qualitative research is needed. This would further the development of frameworks for involving patients and members of the public towards excellence [11]. The aim of this article is to present the findings of a rapid qualitative study carried out with rapid research and evaluation teams around the world to capture their experiences of PPIE in the context of short study timeframes, the challenges they faced, and strategies developed to overcome these. We draw from these experiences to develop the first set of recommendations for PPIE in the context of rapid research and evaluation.

## Methods

The aim of this study was to capture the views and experiences of teams carrying out rapid qualitative research on health-related topics to understand the challenges they have faced while integrating PPIE in study design and implementation and the strategies developed to overcome these. The study was guided by the following research questions:

1. What are researchers’ experiences of patient and public involvement in rapid qualitative studies?
2. How are the views of patients and members of the public integrated in the design and implementation of rapid qualitative research?
3. What are the challenges these teams face?
4. What are the strategies they have used to address these?

### Design

The study was based on a rapid qualitative design using semi-structured interviews to explore current practices and challenges of public and patient involvement in rapid qualitative studies.

### Recruitment and sampling

A snowball sampling approach was used to recruit participants who were working in the field of rapid qualitative research. A list of 90 potential participants was created by the principal investigator (PI), by reviewing the published literature and contacting the authors. The list was then shared with the research lead (RL) who sent the contacts an invitation to participate. The list was then expanded using snowball sampling as the authors who were contacted recommended other researchers working in the field. Recruitment ended for this group once, collectively, contacts had accepted, declined, or did not reply after three invites. In addition, these participants were asked at the end of interviews if they had any appropriate contacts that the research team could approach. The sample included researchers working globally in rapid qualitative studies. Although we acknowledge that this may present differences in governance and research cultures undertaking PPIE work. The inclusion of these researchers was important because of the interest globally in improving PPIE in rapid qualitative studies [19, 20].

A purposive sampling approach was taken to recruit PPIE member participants from networks inclusive of patients, public, carers and service users. PPIE member participants were recruited via two university PPIE networks, one in the South of England and one in the North of England. Both networks supported research teams university-wide and so they were not specific to any particular health condition. The RL contacted the coordinators of each university PPIE network to ask if they would be interested in offering the participation in the study to their PPIE networks. The RL worked with each PPIE coordinator to develop an invitation email which the coordinators then sent out to their PPIE member networks asking for volunteers to take part in an interview, as a participant. The invitations contained the contact details of the RL, for those interested to make direct contact. All potential participants were sent a participant information sheet and consent form and given 48 h to ask any questions and to decide if they would like to take part in the study. All participants provided a signed consent form prior to being interviewed. None of the participants recruited were previously known to the RL who carried out the interviews. Recruitment ended for the PPIE member group once the RL stopped receiving responses from potential participants. Further recruitment rounds were not conducted for either group as data was considered to have reached saturation by 26 interviews. PPIE participants were given a £10.00 gift card as a ‘thank you’ for taking part.

### Inclusion and exclusion criteria

Inclusion criteria

- Member of a PPIE network* with experience of working on rapid projects
- Professionals with experience of working on rapid projects

Exclusion criteria

- Member of a PPIE network* who had not worked on rapid projects
- Professionals who had not worked on rapid projects

*The PPIE networks included patients, public, carers and service users.

### Interviews

Semi-structured interviews were carried out with 15 professionals (herein referred to as “researchers”) working in research, evaluation or PPIE coordinator roles across several institutions and organisations, globally. In addition, 11 patient and public members were interviewed (herein referred to as “PPIE members”). Interviews were carried out over telephone and Microsoft Teams based on participant preference, audio recorded and transcribed by a UCL approved transcription service. Interviews were guided by two topic guides: one for the researchers focusing on their experiences of organising and conducting research with PPIE, and one for the PPIE members focusing on their experiences of being involved in research (please see additional files 1 and 2). Notes were taken during the interviews with main points inputted into a RREAL Rapid Assessment Procedure (RAP) sheet, immediately following interviews [17]. Further recruitment rounds were not conducted as we considered data to have reached saturation by 26 interviews.

### Participant characteristics

Participants were interviewed regardless of their place of work and came from a variety of sectors, including; academic, private, public and charity. Participants who conducted rapid projects either carried out evaluations (work to test effectiveness) or research (work to produce findings) [21]. Table 1 shows a brief description of participant characteristics.

**Table 1:** Participant characteristics

Participant role	England	Scotland	Australia	US	Total
*Type of work*					
PPIE member	10	1			11
Research	5			1	6
Evaluation	5		1		6
PPIE coordinator	3				3
	23	1	1	1	26
*Sector*					
Academic	11				
Private	1				
Public or charity	3				
	15				

### Analysis

The use of RREAL RAP sheets allowed for rapid qualitative methods to be used to analyse the data alongside the collection process by giving real time findings. The data from the RREAL RAP sheets, notes and transcripts were triangulated [17] and analysed using framework analysis to create a matrix of summarised data categories and quotations [22]. The categories for the framework analysis were informed by our research questions and data presenting on the RREAL RAP sheets throughout the study. Once the framework matrix was complete the PI reviewed the data along with the RREAL RAP sheets and consensus on themes was reached. The draft report was sent to the PPIE participants for participant validation. PPIE participants were given one month to voluntarily provide feedback. Individual responses were provided to each participant who provided feedback, with explanation of how their contributions were incorporated into the report.

### Ethical review and governance

The study was approved by the UCL Research Ethics Committee Approval ID Number: (Project number 6862/002), as part of a wider study exploring experiences of research in time-sensitive contexts. Written consent was obtained for all participants in the form of either a signed consent form or email stating the participant consented to take part in the study. These consent approaches were taken as some participants had limited technology skills and were less able to sign an electronic consent form but were able to provide consent by email. All notes and audio recordings were anonymised and stored on a secure UCL server.

## Results

### Defining patient and public involvement and engagement (PPIE)

Both researchers and PPIE members described PPIE as being work which ought to be collaborative, where researchers and PPIE members have equal status and opportunity to contribute to the project. PPIE members considered involvement to be an “opportunity to get a wider and diverse audience to engage with things that actually contribute to, hopefully, improvements and development." (*P.22 PPIE member*), drawing on their lived experience as patients, public, carers and service users. Researchers found PPIE members’ experience was valuable when considering what research to carry out and more specifically, what needed addressing.

“We as researchers, benefit from working collaboratively and openly and transparently with people who use health services. With people who deliver health services. With people who experience health services in different ways. To understand what it’s most appropriate to research and the questions that should be asked about those things” *(P.1 Researcher*).

### Defining rapid research

Both researchers and PPIE members expressed difficulty defining rapid research. For many of the PPIE members, rapid was not a term used specifically to describe PPIE opportunities to them. Most participants (researchers and PPIE members) explained rapid as being where the project needs to start quickly, is carried out in a short timeframe “Just that it needs to be done within much quicker timeframes because the results need to be out there…” (*P.26. PPIE member*) or for time-sensitive public health emergencies “they’ve mostly been about Covid and that’s why they’ve been rapid” (*P.23 PPIE member*). In the case of a longer project, rapid was also experienced as being where findings are delivered throughout the project cycle.

“Rapid can also mean sharing findings early. So, it might be a three-year project, but you might be able to share very formative findings within a matter of weeks or certainly months. You know, so there’s different ways that one might think about rapid” (*P.1 Researcher*).

There are some important distinctions in the perspectives of researchers when comparing the views of those who work in academic settings such as universities with views of workers in the public sector, private consulting firms or charities. For those working in the public, private sector or charities, a rapid approach is considered the normal way of working “I don’t think we ever see it as a thing, as in a particular way of thinking or like your team do. It just happens that some of our work has to be delivered very quickly” (*P.15 researcher*). These participants also suggest that there wasn’t enough understanding that rapid work is “a disciplined area in and of itself. It’s not just doing things fast.” (*P.8 researcher*) and for those working in academic settings, rapid can feel less rigorous “because it’s got to be turned around so quickly, it’s not like an academic schedule, so it may not be as in-depth" (P.7 *Researcher*). However, some felt that what rapid research can deliver is substantial, acknowledging there are some tangible shifts in the mindset of those working in academic settings when thinking about delivering work with speed.

“I actually think what consultancies often do with very limited budgets and very quick turnaround times is very, very impressive. You know, and I think more and more colleagues here, as they experience base work are realising that” (*P1. Researcher*).

### Challenges of integrating PPIE in rapid research

Diversity of PPIE members was the most referenced challenge of integrating PPIE in rapid research. Researchers described PPIE members as being predominantly “retired professionals, probably financially comfortable, educated, etc. and literate with access to devices and Wi-Fi." (P.5 *Researcher*) and would value a wider range of engagement to gain diverse opinions.

“We need a kind of range of people of different age groups, so younger as well as older, which is a real big problem in not having enough younger people and also, kind of people from different walks of life.” (*P.2 Researcher*).

Alongside this, one researcher described being turned down by “a group of a particular minority based at the Student Union” (*P.5 Researcher*) as they received too many requests for involvement work. PPIE members also noted the challenge of diversity with one acknowledging they have “had to ask myself some challenging questions about my own privilege” (*P.25 PPIE member*) and understanding there was an imbalance in the diversity of people who engage in PPIE, realising “the usual suspects” (*P.25 PPIE member*) were often involved.

“I remember being in this focus group once and the chair of it, who was actually a public contributor themselves, saying, “The problem with involving people like you is you know too much,” and you get that a lot, it’s like somehow I am stripped of my patient-ness or my patient experience because I’ve got research literate; it hasn’t taken away my patient experiences it’s just made me very good at holding researchers to account in a way that they don’t like.” (*P.25 PPIE member*).

The nature of rapid research impacts the project setup process and access to PPIE members. For one team, their topics were broad across the healthcare field meaning they felt they would need to recruit new PPIE members for each project. This led to them not carrying out rapid qualitative research with PPIE as time pressures would make PPIE seem tokenistic. While another researcher notes that PPIE members may have other priorities in their life:

“It is their own time and there might be like health issues or other commitments that are sort of have a real impact on their ability to be involved” (*P.3 Researcher*).

The speed in which rapid projects need to start could also make it difficult to recruit the most appropriate PPIE members in time, build relationships with PPIE members and plan to ensure the PPIE is done meaningfully:

“The challenge in the rapid space of course is time, you know, to ensure that patients and the public can contribute on an equal footing and be comfortable in that forum, they often need some induction, some training, some support, some coaching along the way, and we don’t have those people sitting in the wings, so we’d need that lead-in time to do that well in a rapid evaluation setting” *(P.14 Researcher).*

From PPIE members’ perspectives, understanding terminology and research purpose, particularly in short timeframes, could be difficult. PPIE members also expressed not being given much time to review documents, particularly protocols or funding applications where there was a tight deadline for submission:

“They say it’s got to be done very quickly, sometimes because the grant application is going in now, or at the end of the week.” (*P.16 PPIE member*).

### Strategies

To address some of these challenges, research teams either built up their own core group of PPIE members or they were “able to capitalise on existing partnerships” (P.13 *Researcher*) which they had spent time building, to quickly access PPIE members. Other strategies include advertising widely “If you think about it before you do it, then you can target it and make sure you advertise and advertise in such a way that reaches people that you wouldn’t normally reach.” (*P.12 researcher*) and continuing conversations with networks, while being respectful when potential candidates say no. In addition, participants found that being mindful of potential barriers facilitated recruitment of PPIE members.

“So, you're designing in a way that you're enabling participation, so that you are mindful of issues like digital exclusion, like inequalities, like, you know, access to online platforms, all of those issues that affect participation in evaluation” (*P.14 Researcher*).

Other strategies mentioned to reduce barriers to participation in rapid projects were to start planning a PPIE approach as soon as possible, so that once the go-ahead is given, teams know who and how they are going to approach as PPIE members.

“if we’re going to go for these proposal or whatever it is we’d like to involve people, so we need to be thinking now about how to do that” (*P.3 Researcher*).

Researchers placed emphasis on good planning from the outset, especially around setting expectations and boundaries to make sure that PPIE members were aware of the time commitment and timescales. This included being clear on what rapid research meant, for example "the intensity and the frequency of the meetings that we have with PPIE are much quicker and much more frequent" (*P2*. *Researcher*). Further to this, some researchers described planning time into the project timeline for feedback; discussing with the PPIE members how the project was going and ensuring space for measuring PPIE impact and dissemination. Researchers also described integrating PPIE into funding applications, setting research questions, assisting with data collection and analysis, and being involved in dissemination strategies. Most acknowledged that PPIE at every stage would be the ideal, but is not always carried out, with some actively evaluating their own PPIE approaches and methodologies to improve involvement.

“We’re currently undertaking a bit of a review of our PPI work and we’re going to ask PPI members to kind of share their thoughts with us about what they think has worked well” (*P2. Researcher*).

Looking at how teams captured lessons and shared feedback, there was a clear divide between what researchers, and PPIE members experienced. Most researchers expressed capturing lessons either collectively with their team and PPIE members or through self-reflection. In addition, researchers emphasised the importance of giving feedback to PPIE members on how their contribution was used. However, PPIE members reported having limited involvement in capturing lessons following a project; either general project lessons learned or feedback on how their contribution shaped the project. While some did express receiving feedback and finding it helpful, others stated it was not forthcoming, or only provided when they asked PPIE coordinators to follow up with the research teams once the project ended.

“Well, you just feel like, why am I doing this, what contribution, what difference have I made, and it makes me feel quite downhearted, and I don’t think that researchers actually even realise what they’re doing. People take part in research because they want to make a difference, and see that it’s going to make a difference, not only to society but to themselves as well, and with no feedback there’s nothing to support that at all, and a lot of people drop out of research because there’s no feedback.” *(P.24 PPIE member*).

When asked what tools researchers used to carry out rapid qualitative research with PPIE a few said they did not use any ‘formal tools’ or knew of any, but were building up their own toolkit through experience. However, the majority described using RREAL RAP sheets to analyse data alongside data collection, or using large multidisciplinary teams and multiple, experienced, researchers for collaborative analysis. Many also stated using online methods were quicker for rapid projects and the use of digital methods had increased since the COVID-19 pandemic.

### Wider lessons

The overarching advice offered by both groups of participants is to carry out PPIE with meaning and be genuine in the approach so that it is not a tick box exercise. Setting expectations and being clear and transparent with PPIE members around what their involvement will entail and what the timeframes are, was considered key to successful PPIE in rapid qualitative work. In addition, both researchers and PPIE members expressed the importance of treating PPIE members as equal members of the team and providing updates and feedback.

"So authenticity, being genuine. Boundary clear, in your expectations. Clear about timescales, so transparent in your planning and the process" (*P.4 Researcher*).

"PPIE representatives ought to have some kind of equality of status with the other members of the research team, while at the same time, bringing a kind of unique or certainly a different perspective…" (*P.20 PPIE member*).

Researchers considered existing PPIE networks to be a facilitator as well as having PPIE members who understand the nature of rapid work "Another facilitator especially has been kind of, because our PPIE members understand that we’re doing rapid evaluation and that we’ve explained it to them quite clearly" (*P.2 Researcher*). What further helps PPIE members get up to speed with a new rapid project is having clear descriptions of the project using plain language and given in a timely manner so that they can understand the purpose of the research quickly. In addition, PPIE members have found training on patient involvement to be useful, whether for rapid or non-rapid research. They also found benefit in buddying up with experienced PPIE members, public speaking and interview skills training and qualitative and quantitative training.

The benefits of rapid qualitative training were recognised by PPIE members, which involved adopting specific skills and ways of working such as focusing on what matters most and undertaking data collection and analysis in parallel. PPI members expressed the value of this training and how it can be applied to other projects more widely.

“One of my take home messages was the advantage of starting different components of a study concurrently. I could see this was a distinct benefit of rapid qualitative research. It was a valuable training course for me and I've referred back to it during progression of a number of projects” *(P.23 PPIE member).*

However, there were inconsistencies in PPIE members’ experiences of being offered training, with many not being asked what training needs they had. These participants felt training on the following would be beneficial: qualitative analysis, updated PPIE methods, reviewing applications, how to track changes and understanding systematic reviews. In addition, although training is available from some organisations, PPIE members enthusiasm and commitment were often the reasons why they sought training and continued their PPI work.

“I have always been proactive in seeking training to support my PPI. I believe this is the reason I’ve progressed” *(P.23 PPIE member).*

## Discussion

In this study, we aimed to explore the experiences of PPIE in teams carrying out rapid qualitative research, including the challenges they faced, and the strategies they used to address these. Researchers and PPIE members expressed a view that PPIE should be an equal and collaborative approach, similar to what has been reported in the literature for non-rapid research [4, 23]. There was also a strong message that PPIE must be genuine, underpinned by honesty and clear expectations [24]. However, in the present study focusing on rapid research, there was a misalignment between how participants perceived PPIE ‘ought’ to be carried out and how they described PPIE ‘was’ carried out. Many of the researchers described involving PPIE members throughout the project cycle, where possible. Whereas the PPIE members interviewed had predominantly worked on the beginning stages of research, mostly reviewing protocols and funding applications with few examples of being involved in data collection, analysis, dissemination or reflecting on lessons learned. Further to this, PPIE members stressed that there was a lack of consistent feedback on how their contributions were used by researchers.

Similar to studies on non-rapid research and PPIE [6], we found that recruiting a diverse PPIE member group was identified as a challenge by both researchers and PPIE members. However, increasing diversity among PPIE members should not be done at the expense of those already taking part [5]. Researchers in the present study have attempted to overcome this by trying not to over-use the same PPIE members and actively being mindful of barriers for hard-to-reach, seldom heard populations. However, this should not be solely the researcher’s responsibility. As Reynolds et al. [5] argue, researchers need a PPIE infrastructure in place which supports diverse recruitment of PPIE members. Suggesting better financial process for PPIE reimbursement, well-supported PPIE coordinators and less researcher turnover within projects, all to be done with balance so as not to compromise existing relationships with PPIE members who are more easily able to participate [5].

Moreover, the participants of this study highlighted that access to PPIE members can be complicated when working with time pressures. PPIE members reflected on the challenges of responding to quick turnaround times for protocol and funding application reviews. The speed at which research can now be set up may pose further challenges around diversity, due to having less time to recruit from a wider range of populations. Similarly, to what participants in the present study have indicated, a recent report also found young people and ethnically diverse communities are the most underrepresented in PPIE. The majority of PPIE members in research tend to be older, white British and female [25]. The commonly reported reasons for this are language barriers, mistrust, financial loss due to participation, limited flexibility of PPIE members, and complex study designs [25]. Researchers in the present study often use tools specifically designed for rapid approaches, such as RREAL RAP sheets [17] for multi-researcher analysis and data collection carried out in parallel to each other. In addition, researchers expressed finding online communication quicker than face to face.

Rapid approaches and techniques call for further understanding of timeframes and specific training. As such, rapid approaches may pose additional burdens on PPIE members, and further narrowing the scope for diversity compared to traditional research methods. These are not new findings around PPIE. Many studies have reported a lack of PPIE in the latter stages of research [26], inconsistent training [27] little feedback [28] and difficulty recruiting diverse groups [29]. However, we can now demonstrate that these barriers also apply to rapid research which is especially problematic when working in time-sensitive contexts.

A common suggestion from participants when asked what facilitated PPIE in rapid qualitative research was to have an existing network of PPIE members set up, who are familiar with rapid methods and responsive to rapid requests [30]. However, this may also increase the issue of repetitively recruiting the same people. Rapid researchers can overcome issues where small numbers of motivated and trained PPIE members are involved by building relationships with wider groups of PPIE members in an ongoing way, such as increasing attendance at support group meetings and working with health charities. This will increase opportunities for networking and the ability of researchers to have a pool of relevant people to draw from.

A particular challenge for PPIE members was understanding the research purpose when jargon and non-plain language was used. As in the present study, jargon was often seen as a barrier to PPIE members being able to quickly get to grips with what research was aiming to achieve [31]. Providing resources and communicating what we do in plain language actively seeks more diversity and reduces inequities of access [32]. Creating research documents using language that is easily  understood offers opportunities for wider audiences to become involved and may speed up PPIE work. In addition, involving PPIE members in the dissemination can help findings be more understandable and, therefore, accessible to the general population [33]. Other approaches such as meeting people one to one, or where appropriate face to face so that researchers may meet quickly and informally to discuss matters of urgency and provide additional support can help address the challenges of meeting the needs of PPIE members and researchers.

The published evidence has highlighted that it is important to take the time to find out what skills PPIE members have to facilitate their involvement [34]. In the case of our study, some PPIE members already had experience in research or had transferrable skills from previous work or volunteering roles. However, it is important to note that although PPIE members may be involved in previous work they may not have relevant experiential knowledge to contribute to current work. In addition, we found training was not consistent across PPIE members; they did not seem to be offered training routinely or asked if they felt they would benefit from any training [35]. We also found variation in the training on PPIE carried out by researchers and, this study identifies the need for training for both researchers and PPIE members which is common to all involvement in relation to increasing diversity, being responsive to PPIE members and the need for clearer guidelines [8]. Whilst other studies have demonstrated that training researchers on the practicalities of working with PPIE members has shown to increase confidence and likelihood of involving patients and the public in research [36] this is not always common practice, and the training did not include strategies for rapid research.

### Study limitations

The grouping of participants under a collective term is contentious given the lack of homogeneity across patients, public, carers and service users, which fails to account for differences within groups [37]. The present research accepts the inherent problem in the ‘PPIE’ term, however as per NIHR INVOLVE [1]the present study considers patients, carers and service users as members of the public. We include the use of this term as they are related areas which impact health research [20]. PPIE activities do not always cater to the degree of diversity within its members, unless there is a specific call i.e., patients required for a specific trial. We focus on commonalities within the PPIE members’ experiences, such as inclusion and greater ability to impact health research.

When listening to participants, there was a sense that, in some cases, they may have been referring to their PPIE experiences in general and not always in the context of rapid research. Participant characteristics showed that of the 26 participants, three were unsure when asked if they had experience of rapid qualitative research (one researcher and two PPIE members) and one participant stated they had no experience of rapid qualitative research (PPIE member). Two participants (both researchers) were in the beginning stages of their first rapid projects, which meant some researchers were less experienced. Nevertheless, this was known from the outset and these participants were able to contribute to the discussion by exploring what their perceptions and plans were for carrying out rapid qualitative research with PPIE members.

Limitations around recruitment were that we only sourced PPIE members from two PPIE networks, however, data saturation had been reached by 11 PPIE member interviews. Although it is acknowledged that recruiting from a third PPIE network may have yielded new data, the present study is an early-stage piece of research which is going toward a larger project to co-produce a model for carrying out PPIE in time-sensitive contexts. As such, a data collection line was drawn at 26 interviews. A further limitation was the geographical spread of participants. While the research team, for the present study, attempted to recruit participants globally, there were limited responses from potential participants outside of England. As such, all but three of the participants were located in England, with one in Scotland, one in the US and one in Australia. Lastly, all documents and interviews were in English, limiting the voice of non-English speakers in the research.

The use of existing frameworks and checklists are available such as the Guidance for Reporting Involvement of Patients and the Public (GRIPP2) checklist [38]. The GRIPP2 checklist was utilised in this study to improve the quality, transparency, and consistency of PPIE reporting (please see Additional file 3). However, the GRIPP2 checklist does not identify the extent of PPIE involvement in studies identified as rapid.

### Implications & recommendations

This study raises awareness about the challenges of implementing PPIE in rapid research, and the future opportunities for PPIE within shorter timeframes. Rapid qualitative researchers endorse PPIE and would benefit from following frameworks and checklists which provide instructions of the rapid process of design, analysis, and dissemination of PPIE research. This study was the first to describe and set priorities for rapid research projects. This can be done by integrating more PPIE so that it becomes the norm, not the exception, and define rapid from the outset of each project. Researchers should use PPIE models based on diverse groups of PPIE members who can be recruited rapidly, while maintaining relationships with existing contributors. To achieve a co-creation process there should be basic training for PPIE members on PPIE approaches and rapid qualitative research methods, and basic training for researchers on the benefits of PPIE. This study found that researchers need to know how to recruit and maintain a reliable PPIE network and how to best utilise their existing skills and experience. Researchers should plan time for feedback to PPIE members on both their ‘performance’ and how their contribution shaped the project which would contribute to higher quality PPIE in future projects.

## Conclusion

In this study, we found that patients, public, carers and service users can be actively involved in rapid qualitative research, despite tangible time pressures. The early identification of training needs can assist both researchers and PPIE members who desire to integrate PPIE in rapid research. We also found that providing feedback on members’ contribution to shaping knowledge and creating social change were important for engagement with rapid qualitative projects. This study facilitated understanding of the reasons usually offered for a lack of participation in PPIE research and many of the challenges and strategies discussed in this paper can be applicable to research carried out in a longer timeframe. However, in times of increased demand for rapid results it is prudent to have a better understanding of the nuances these challenges impose of PPIE in rapid research so that appropriate strategies can be developed and implemented. Future areas of research and the application of these findings include the development of strategies to involve PPIE members in the rapid interpretation of findings and dissemination as well as the creation of tools to rapidly assess the impact of PPIE members’ involvement in research (Additional file: 1, 2 and 3).

## Supplementary Information

**Additional file 1.** Interview topic guide for researchers.

**Additional file 2.** Interview topic guide for PPIE members.

**Additional file 3.** GRIPP 2 short form.

## Data Availability

The datasets used and/or analysed during the current study are available from the corresponding author on reasonable request.

## Abbreviations

PPIE: Patient and public involvement and engagement
RREAL: Rapid Research Evaluation and Appraisal Lab
RAP: Rapid appraisal procedure
GRIPP2: Guidance for reporting involvement of patients and the public
