# Supplementary material for: The role of patient and public involvement in rapid qualitative studies: Can we carry out meaningful PPIE with time pressures?
Source: Res Involv Engagem. 2022 Nov 30;8:67. doi: 10.1186/s40900-022-00402-5 (PMC9713187; doi:10.1186/s40900-022-00402-5)
Supplement: Supplementary file 1 — Additional file 1. Interview topic guide for researchers. [file 40900_2022_402_MOESM1_ESM.docx]

Interview topic guide for Researchers:

The role of patient and public involvement in rapid qualitative studies: Can we carry out meaningful PPIE with time pressures?

***Interviewee role***

1. I was wondering if we could begin with a description of your current role.
2. How long have you been in this role?

***Interviewee team***

1. Could you give a description of the team you work with?
   1. How big is the team?
   2. Are the members FT/PT
   3. What department does the team sit under?
   4. Is it a multi-disciplinary team? (anthropologists/sociologist/clinicians)
   5. How long has the team been together?
   6. How is the team funded?

***Type of research***

1. What are the main research topics of the team?
2. How does the team decide what work to take on?
   1. How do you source work?
   2. Do you bid for funding?
   3. Consultancy?
3. What are the main types of research undertaken? (Qual/quant/MM)
4. Can you give me an idea of how you split the resources (people and funding) across different types of research?

***Rapid qualitative research***

1. Could you tell me what Rapid qualitative research your team carries out?
2. Could you tell me what Rapid tools you use?

***PPIE***

1. How would you define patient and public involvement?
2. How is rapid research explained to and understood by patients and public?
3. How are the views of patients and members of the public integrated?
   1. Design?
   2. Implementation?
   3. Dissemination?
   4. Do these strategies differ with non-rapid research?
4. What are the challenges of including PPIE into rapid research?
5. How have you addressed these challenges and if they were not able to be addressed, what do you think would have helped?
6. What are the facilitators of including PPIE into rapid research?

***Development***

1. How do you capture lessons learned following a rapid study with PPIE?
2. How have your strategies developed/improved for approaching and carrying out rapid research with PPIE?
3. Do you have any advice for other teams who may be considering a rapid study with PPIE?
