# Supplementary material for: The role of patient and public involvement in rapid qualitative studies: Can we carry out meaningful PPIE with time pressures?
Source: Res Involv Engagem. 2022 Nov 30;8:67. doi: 10.1186/s40900-022-00402-5 (PMC9713187; doi:10.1186/s40900-022-00402-5)
Supplement: Supplementary file 2 — Additional file 2. Interview topic guide for PPIE members. [file 40900_2022_402_MOESM2_ESM.docx]

Interview topic guide for PPIE members:

The role of patient and public involvement in rapid qualitative studies: Can we carry out meaningful PPIE with time pressures?

***Interviewee role***

1. I was wondering if we could begin with when and how you became a PPIE member?
2. How often do you take part in PPIE work?

***Type of research***

1. What are the main research topics you have worked on?
2. What kind of tasks might you be offered to contribute to?
3. How do you decide which tasks to accept?
4. Which type of tasks do you enjoy the most?
5. Do you have a preference of working on qualitative or quantitative projects?

***Rapid qualitative research***

1. Could you tell me about any Rapid qualitative tasks you have completed?
2. How have these tasks differed to tasks where the study was not considered rapid?

***PPIE***

1. How would you define patient and public involvement?
2. As a PPIE member, how is rapid research explained you?
3. Have you received any training on being a PPIE member?
4. Is there any training you think you would have benefited from having?
5. How are your views as a PPIE member integrated into the work?
   1. Design?
   2. Implementation?
   3. Dissemination?
6. What are the challenges of contributing to rapid research?
7. How have you addressed these challenges and if they were not able to be addressed, what do you think would have helped?
8. What are the facilitators of contributing to rapid research?

***Project wrap up***

1. Have you been involved in any kind of project wrap up tasks such as meeting to discuss any lessons learned? If so, could you describe these experiences?

1. Where you are completing a task such as a review of documents, do you hear feedback regarding your contributions?
2. What have you personally learned from being a PPIE member?
3. Do you have any advice for research teams who may be considering a rapid study with PPIE?
4. Do you have any advice for people considering becoming a PPIE member?
5. If you could change anything about you experiences as a PPIE member, what would that be?
6. Do you have anything additional to add?
