# Supplementary material for: The role of patient and public involvement in rapid qualitative studies: Can we carry out meaningful PPIE with time pressures?
Source: Res Involv Engagem. 2022 Nov 30;8:67. doi: 10.1186/s40900-022-00402-5 (PMC9713187; doi:10.1186/s40900-022-00402-5)
Supplement: Supplementary file 3 — Additional file 3. GRIPP 2 short form. [file 40900_2022_402_MOESM3_ESM.docx]

GRIPP2 short form

| Section and topic | Item | Reported on page No |
| --- | --- | --- |
| Aim | Report the aim of PPI in the study | Page 2, 4 |
| Methods | Provide a clear description of the methods used for PPI in the study | Page 4-7 |
| Study results | Outcomes—Report the results of PPI in the study, including both positive and negative outcomes | Page 7-15 |
| Discussion and Outcomes | Comment on the extent to which PPI influenced the study conclusions overall. Describe positive and negative effects | Page 15-17 |
| Reflections/critical perspective | Comment critically on the study, reflecting on the things that went well perspective and those that did not, so others can learn from this | Page 17-18 |

PPI patient and public involvement
